# Effectiveness of psychosocial and therapeutic interventions for children and adolescents with adverse childhood experiences: A systematic review and meta‐analysis

**DOI:** 10.1002/jcv2.70186

**Published:** 2026-11-04

**Authors:** Meless G. Bore, Julaine Allan, Setognal B. Aychiluhm, Kedir Y. Ahmed, Abel Fekadu Dadi, Elsa Awoke Fentie, Subash Thapa

**Affiliations:** ^1^ Rural Health Research Institute Charles Sturt University Orange New South Wales Australia; ^2^ College of Medicine and Health Sciences Hawassa University Hawassa Ethiopia; ^3^ Division of Maternal and Child Health, Menzies School of Health Research, Hearing and Ear Health Charles Darwin University Darwin Northern Territory Australia; ^4^ Translational Health Research Institute Western Sydney University Campbelltown Campus Campbelltown New South Wales Australia; ^5^ Discipline of General Practice, School of Medicine Adelaide University Adelaide South Australia Australia; ^6^ Addis Continental Institute of Public Health Addis Ababa Ethiopia; ^7^ Centre for Child Development and Education Menzies School of Health Research Casuarina Northern Territory Australia; ^8^ School of Population Health Faculty of Medicine and Health Science University of New South Wales Sydney New South Wales Australia

**Keywords:** Adverse Childhood Experiences, anxiety, children and adolescents mental health, depression, mental health, meta‐analysis, personalized intervention, trauma‐informed care

## Abstract

**Background:**

Adverse Childhood Experiences (ACEs) are important risk factors for a range of mental health conditions among children and adolescents. This meta‐analysis examined the effectiveness of psychosocial and therapeutic interventions on anxiety, depression, post‐traumatic stress disorder (PTSD), and psychosocial functioning among children aged ≤18 years with documented ACE exposure.

**Methods:**

We systematically searched randomized controlled trials (RCTs) published between January 1, 2000 and September 17, 2026 that evaluated psychosocial and therapeutic interventions and reported relevant mental health outcomes among children and adolescents with documented ACE exposure. Searches were conducted in eight electronic databases (PubMed, MEDLINE [Ovid], Embase, PsycINFO, CINAHL, Cochrane Library, Web of Science, and EBSCOhost). Study quality was assessed using the Cochrane Risk of Bias 2.0 tool. Pooled effect sizes (Hedges' *g*) were estimated using random‐effects meta‐analysis. Subgroup analyses were conducted by intervention modality, duration, setting, country income level, and reported ACE exposure characteristics.

**Results:**

After duplicate removal, 6087 records were screened, and 21 RCTs involving 2450 children from nine countries were included. Eleven studies were found to have low risk of bias, and 10 had some concerns, with no studies assessed as high risk of bias. Meta‐analysis of six RCTs showed that cognitive behavioral therapy (CBT), trauma‐focused and resilience‐building interventions significantly reduced anxiety symptoms (Hedges' *g* = −0.46; 95% CI −0.86 to −0.06). Particularly, trauma‐focused interventions demonstrated significant reductions in anxiety in subgroup analyses (Hedges' *g* = −0.90; 95% CI −1.66 to −0.14). Meta‐analysis of four RCTs indicated that CBT and resilience‐building interventions lasting ≥8 weeks significantly reduced depressive symptoms (Hedges' *g* = −0.26; 95% CI −0.44 to −0.07). No significant pooled or subgroup effects were observed for PTSD or broader psychosocial outcomes.

**Conclusion:**

CBT‐based, trauma‐focused and resilience building interventions effectively reduce anxiety and depressive symptoms among children with documented ACE exposure. Integrating evidence‐based psychological interventions with trauma‐informed principles may strengthen the delivery of mental health care for children and adolescents with documented ACE exposure and mental health needs.

## Key Points

**What's known?**

- Adverse Childhood Experiences (ACEs) increase the risk of anxiety, depression, post‐traumatic stress disorder (PTSD), and impaired psychosocial functioning in children and adolescents.
- Psychosocial and therapeutic interventions, particularly cognitive behavioral therapy (CBT), have been suggested as effective strategies but evidence across multiple ACE‐exposed populations has been limited.

**What's new?**

- This meta‐analysis of 21 randomized controlled trials (2450 children) found that CBT, including trauma‐focused and resilience‐building interventions, significantly reduces anxiety and depressive symptoms, particularly when interventions last ≥8 weeks.
- Trauma‐focused approaches alone also reduce anxiety. No significant effects were observed for PTSD or broader psychosocial outcomes.

**What's relevant?**

- Findings provide evidence supporting the use of structured CBT and trauma‐informed resilience interventions in clinical and educational settings for children and adolescents with documented ACE exposure and mental health needs.
- The results can inform policy and service delivery by highlighting evidence‐based approaches that may reduce anxiety and depressive symptoms among children and adolescents with documented ACE exposure.

## BACKGROUND

Adverse Childhood Experiences (ACEs) are potentially traumatic experiences/events that occur before the age of 18. These traditionally include physical, sexual, or emotional abuse; physical or psychological neglect; and household dysfunctions such as domestic violence, parental incarceration, substance abuse, familial mental illness, or parental separation (Bellis, 2016). More recent conceptualizations also include socio‐environmental adversities, such as bullying and extreme weather events (Thapa et al., 2025). ACE exposure is consistently associated with an increased risk of adverse developmental, emotional, behavioral, and mental health outcomes across the life course, with cumulative exposure conferring progressively greater risk (Bellis, 2016; Giri et al., 2025).

ACEs and trauma are related but distinct constructs. ACEs refer to specific categories of potentially adverse childhood exposures (e.g., abuse, neglect, household dysfunction), whereas trauma refers to the psychological and physiological response an individual may experience following exposure to a distressing event, which not all children with ACE exposure will develop (Bellis, 2016; J. World Health Organization, 2018). Conversely, trauma responses can arise from experiences not captured within standard ACE framework. Regardless of whether a trauma response develops, children and young people exposed to ACEs face a heightened risk of developing mental health conditions, including depression, anxiety, post‐traumatic stress disorder (PTSD), and a range of trauma‐related behavioral and emotional problems, substance use disorders, and suicidal ideation (Fuller‐Thomson et al., 2016; Leza et al., 2021; Merrick et al., 2017). Among these, complex PTSD, as defined in ICD‐11, is particularly associated with prolonged or repeated interpersonal trauma, including childhood abuse and neglect, and involves additional disturbances in self‐organization beyond core PTSD symptoms (J. World Health Organization, 2018).

A range of psychosocial and therapeutic interventions, such as CBT, mindfulness‐based approaches, resilience‐building programs and integrated psychosocial programs, have been used to address mental health and psychosocial difficulties among children and adolescents. Some of these interventions have demonstrated promising effects in reducing trauma‐related symptoms and improving emotional and behavioral functioning (Murphy et al., 2023; Thielemann et al., 2022), whereas others have reported mixed or limited evidence of effectiveness (Lee & Rawlings, 2022; Sanders et al., 2020).

In response to the complex and diverse risks associated with childhood adversity and trauma exposure, many interventions increasingly incorporate trauma‐informed approaches, such as trauma focused‐CBT (TF‐CBT), family‐centered therapy models, and traditional healing–integrated programs, which aim to improve engagement, cultural responsiveness, and sustained mental health outcomes (Cohen et al., 2004; Goldbeck et al., 2016; Gone et al., 2019; Goodkind et al., 2012; Jongen et al., 2023; Kameoka et al., 2020; Thielemann et al., 2022). Previous studies have shown that such trauma‐focused and individualized care models can reduce anxiety and stress symptoms among children and young people (Goldbeck et al., 2016; Kameoka et al., 2020). Evidence also highlights the potential of digital and hybrid trauma‐informed interventions, such as telehealth‐delivered TF‐CBT and digitally supported trauma interventions, to expand access to care, especially in rural and underserved settings (Martin et al., 2023; Stewart et al., 2017). However, variation in intervention effectiveness, limited mental health workforce capacity and implementation barriers such as resource constraints continue to limit the availability and scalability of trauma‐informed interventions in many settings (Roundfield & Lang, 2017).

Even in settings where interventions and services are available, supporting children and adolescents with histories of chronic stress or trauma exposure can present clinical challenges, including difficulties with treatment engagement, therapeutic trust, and continuity of care (Asseffa et al., 2026). ACE exposure has been associated with greater emotional and behavioral difficulties, interpersonal difficulties, and higher levels of mental health comorbidity and service needs (Nelson et al., 2020). Children presenting with similar mental health conditions may require different clinical considerations depending on their trauma history and psychosocial context. According to the U.S. Substance Abuse and Mental Health Services Administration, a program, organization, or system that is trauma‐informed “realizes the widespread impact of trauma and understands potential paths for recovery; recognizes the signs and symptoms of trauma in clients, families, staff, and others involved with the system; and responds by fully integrating knowledge about trauma into policies, procedures, and practices, and seeks to actively resist re‐traumatization” (Substance Abuse and Mental Health Services Administration, 2014). Trauma‐informed interventions therefore aim to address the broader consequences of trauma exposure by promoting safety, empowerment, and recovery, rather than focusing solely on specific trauma‐related diagnoses.

However, many existing trauma‐focused intervention studies have primarily targeted children with clinically diagnosed PTSD or trauma‐related disorders in clinical settings, often involving small samples, rather than children and adolescents with documented ACEs who may present with a broader range of mental health difficulties, and who may or may not meet criteria for a trauma‐related disorder. Furthermore, several studies have evaluated single outcomes, limiting understanding of intervention effects across multiple domains of child and adolescent mental health. At the evidence synthesis level, previous systematic reviews have similarly focus predominantly on trauma‐related disorders or PTSD‐specific interventions among clinically diagnosed populations (Gone et al., 2019; Lorenc et al., 2020; Murphy et al., 2023; Smith et al., 2019; Thielemann et al., 2022).

Despite increasing recognition of the importance of integrating trauma history into mental health assessment and intervention delivery, the overall effectiveness of psychosocial and therapeutic interventions among children and adolescents with documented ACE exposure remains unclear. To our knowledge, no meta‐analysis has synthesized the pooled effects of these interventions on anxiety, depression, PTSD symptoms, and psychosocial outcomes in this population. Therefore, this systematic review and meta‐analysis investigated the effectiveness of psychosocial and therapeutic interventions in reducing symptoms of anxiety, depression, and PTSD, and improving psychosocial functioning among children and adolescents aged ≤18 years with documented ACE exposure.

## METHODS

This systematic review and meta‐analysis was reported based on the Preferred Reporting Items for Systematic Reviews and Meta‐Analyses (PRISMA 2020 statement) guidelines (see Supporting Information S1: Appendix S1) (Page, 2021).

### Eligibility criteria

The research question was: among children and adolescents aged ≤18 years with documented exposure to ACEs and diagnosed mental health disorders, clinically elevated symptoms, or identified psychosocial difficulties, what are the effects of psychosocial and therapeutic interventions on mental health and psychosocial outcomes, compared with standard care, treatment as usual, wait‐list controls, or non‐trauma‐focused services? Eligibility criteria were developed using the population, intervention, comparison, and outcome framework to structure the research question, as summarized in Table 1. We included randomized controlled trials (RCTs) and cluster‐RCTs, published in English from January 2000 onward. This period reflects the widespread adoption of the ACE framework in child mental health research and the subsequent emergence of contemporary intervention models and standardized diagnostic and outcome‐assessment practices. Studies were eligible if they used validated, standardized self‐report or proxy‐report (parent/caregiver‐ or clinician‐rated) outcome measures, while studies relying solely on non‐validated, experimental, or observational measures were excluded.

**TABLE 1:** PICO framework.

PICO element	Criteria
Population (P)	Children and adolescents aged ≤18 years with documented ACE exposure (abuse, neglect, household dysfunction, or other potentially traumatic childhood adversities); ACE exposure treated as a contextual factor, not an indication for intervention. Included participants with diagnosed mental health disorders (depression, anxiety, PTSD, other trauma‐related disorders), clinically elevated symptoms, or identified psychosocial difficulties following ACE exposure.
Interventions (I)	Psychosocial interventions (e.g., family therapy, parenting programs, peer support, resilience‐building) aimed at strengthening family relationships, coping, social support, and adaptive functioning; and therapeutic interventions (e.g., CBT, DBT, ACT, MBCT, IPT) aimed at reducing psychological symptoms and improving emotional regulation, coping, and interpersonal functioning.
Comparators (C)	Standard care, treatment as usual, wait‐list controls, or non‐trauma‐focused services, as defined by the original studies (routine clinical, educational, or community‐based services).
Outcomes (O)	Mental health and psychosocial outcomes: Reductions in depression, anxiety, PTSD, and trauma‐related symptoms; improvements in resilience, self‐esteem, coping, social support, quality of life, educational outcomes, and overall functioning.

Abbreviations: ACE, Adverse Childhood Experience; ACT, acceptance and commitment therapy; C, comparator; CBT, cognitive behavioral therapy; DBT, dialectical behavior therapy; I, intervention; IPT, interpersonal psychotherapy; MBCT, mindfulness‐based cognitive therapy; O, outcome; P, population; RCT, randomized controlled trial.

### Information sources and search strategy

We performed comprehensive search across eight electronic databases: PubMed, MEDLINE (Ovid), Embase, PsycINFO, CINAHL, Cochrane Library, Web of Science, and EBSCOhost for studies published between January 1, 2000 and September 17, 2026. Searches were initially conducted on January 9, 2026 and updated on September 17, 2026 to capture recent studies published in the interim. Additionally, Google Scholar and manual screening of reference lists of identified articles were conducted.

The search strategy used Medical Subject Headings, database‐specific controlled vocabulary, and keywords relating to the population, interventions, and study design (see Supporting Information S1: Appendix S2). To enhance sensitivity and minimize the risk of missing relevant studies, no restrictions were applied to comparators or outcomes during the search process. All searches were developed and refined in consultation with the research team.

### Study selection

All retrieved records were imported into Covidence systematic review software for management and screening. Two reviewers (M.G.B and E.A.F) independently screened titles and abstracts for eligibility. Disagreements were resolved through discussion and consensus or, when necessary, through consultation with a third reviewer (S.T.). Full‐text articles were then independently assessed for eligibility by the same two reviewers, with disagreements resolved in the same manner. Studies that did not meet inclusion criteria were excluded, and reasons for exclusion were recorded.

### Data extraction

A standardized extraction template, adapted from the Cochrane Handbook for Systematic Reviews of Interventions (Higgins et al., 2019), was used. Two reviewers (M.G.B and E.A.F) independently extracted data, which were cross‐checked for accuracy. Extracted data included author, year of publication, country, study design, participant characteristics (age, sex, ACE categories and cumulative exposure, mental health condition), intervention details (type, duration, frequency, cultural adaptation, and comparator details), outcomes measured (mental health and psychosocial domains), and follow‐up periods and results. For studies with incomplete data, corresponding authors were contacted twice. Where no response was received, only available data were analyzed.

### Risk of bias

Two reviewers (M.G.B and K.Y.A) independently assessed the risk of bias in the included studies. Cochrane Risk of Bias 2.0 tool was applied to evaluate the methodological quality of the included trials, which examines five key domains: (i) randomization procedures, (ii) deviations from intended interventions, (iii) missing outcome data, (iv) outcome measurement, and (v) selective reporting of results (Sterne et al., 2019). Each study was assigned an overall judgment of low risk of bias, some concerns, or high risk of bias based on these assessments. Discrepancies were resolved by discussion.

### Statistical analysis

All analyses were conducted using R version 4.3.3. For continuous outcomes (anxiety, depression, PTSD symptoms, and psychosocial outcomes), post‐intervention endpoint means and standard deviations (SDs) were extracted and used for meta‐analysis, rather than mean change scores. Change‐score statistics (and the pre‐post correlations needed to estimate their SDs) were not consistently reported across included studies. Baseline values were not formally compared or statistically adjusted for. As all studies included were RCTs, baseline comparability between arms was expected through randomization, and the quality of the randomization process was assessed as part of the risk‐of‐bias assessment. Where means and SDs were not directly reported, values were imputed from reported standard errors, 95% confidence intervals, or *p*‐values following Cochrane guidelines (Higgins, 2024). Where studies reported multiple follow‐up time points, the primary post‐intervention assessment was prioritized to maximize comparability across studies.

Effect sizes were calculated as Hedges’ *g* with 95% confidence intervals. Hedges’ *g* was estimated using post‐intervention means and SDs from intervention and control groups with the *metacont()* function in the *R* meta package. The between‐group difference in post‐intervention scores was standardized using the pooled post‐intervention SD, with correction for small‐sample bias. Risk ratios (RR) were pre‐specified for dichotomous outcomes. However, no included studies reported outcomes in a format amenable to RR pooling, and all effect sizes presented are therefore Hedges’ *g*.

Given heterogeneity in study designs and interventions, a random‐effects meta‐analysis (inverse‐variance method) was applied. Heterogeneity was assessed with the *I* ^2^ statistic. Separate meta‐analyses were conducted for mental health outcomes (depression, anxiety, psychological distress and PTSD). Interventions were pooled together in the primary analyses (e.g., CBT, trauma‐focused, and resilience‐building approaches) due to the limited number of studies available for each individual intervention type and substantial clinical overlap between categories. Intervention‐specific effects were explored separately in subgroup analyses by modality. Forest plots were generated to visually display individual and pooled effect sizes. Subgroup analyses were performed by intervention duration, intervention settings and country context. Publication bias assessment (e.g., funnel plots, Egger's test) was planned but not conducted because fewer than 10 studies contributed to each meta‐analysis, consistent with established guidelines (Higgins, 2023; Jakobsen et al., 2014).

## RESULTS

### Study characteristics

We identified 6217 articles (6205 from databases and 12 from citation searching). After removing 125 duplicates, 6087 records underwent title and abstract screening, with 5639 excluded. A total of 448 full‐text articles were sought, of which 9 were not retrieved. The remaining 439 full texts were screened, and 415 were excluded. Finally, 21 RCTs from nine countries were included in the analysis (Figure 1). Most studies were conducted in the United States (*k* = 10, 47.6%), followed by China (*k* = 3, 14.3%). Single studies were identified from Burundi, Rwanda, Australia, Norway, the Netherlands, Japan, Mexico, and South Africa (each representing 4.8%). Based on the World Bank income classifications, most studies were conducted in high‐income countries (*k* = 14, 66.7%), followed by upper‐middle‐income countries (*k* = 5, 23.8%), and low‐income countries (*k* = 2, 9.6%).

**FIGURE 1:**
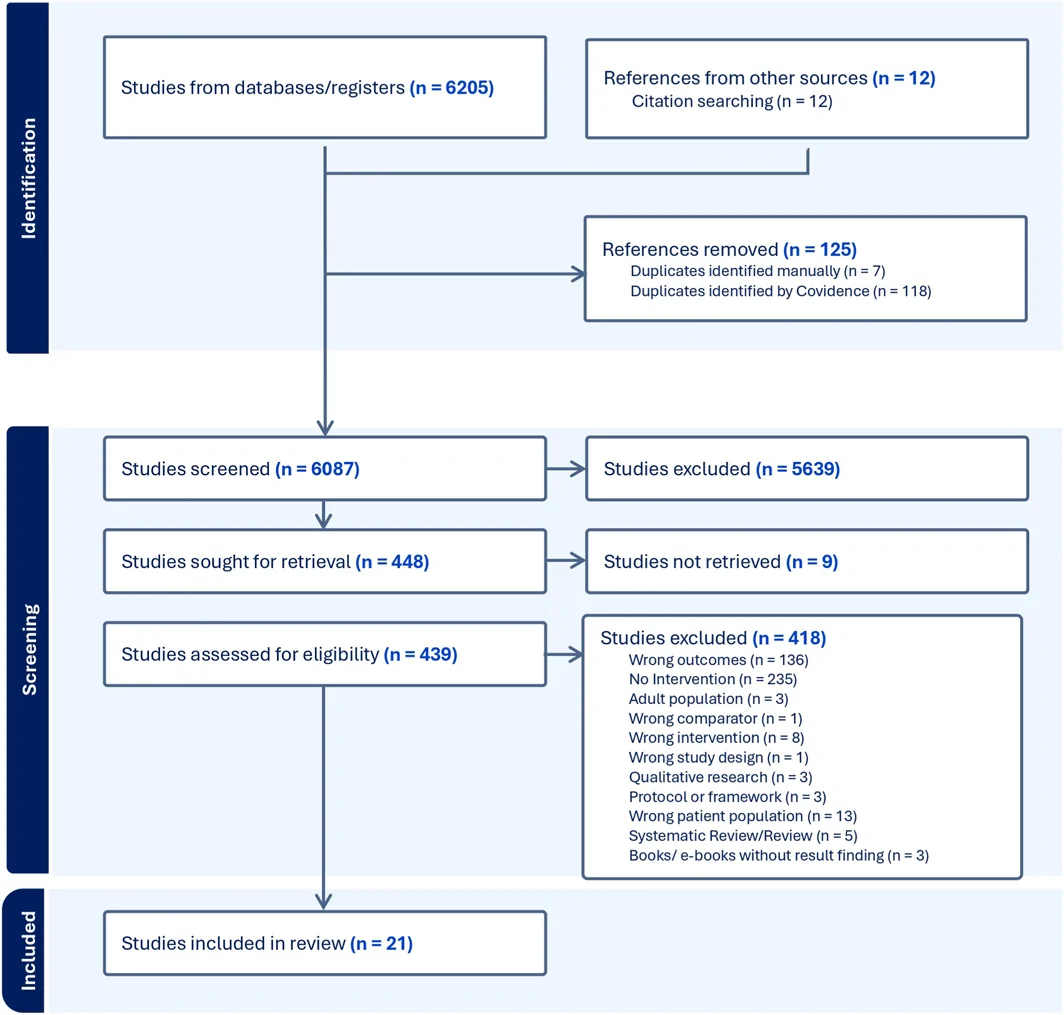
PRISMA flow diagram.

The 21 RCTs included in this review involved a total of 2450 participants (age ranges from 2 to 18 years), with 1208 in the intervention group (IG) and 1242 in the control group (CG). The sample sizes varied across outcomes, as several studies reported more than one outcome. Depression was assessed in 1883 participants (926 in the IG and 957 in the CG), anxiety in 327 participants (169 in the IG and 158 in the CG), and PTSD in 703 participants (348 in the IG and 355 in the CG). Mixed anxiety–depression was reported for 206 participants (112 in the IG and 94 in the CG), while psychosocial problems were evaluated in 1399 participants (679 in the IG and 720 in the CG).

Across 21 trials, the median intervention duration was 8 weeks (IQR 6–10; range 3–42), with 14/21 (66.7%) lasting ≥8 weeks. Among the included therapeutic interventions, CBT and its variants were the most common (8/21; 38.1%) (Brent et al., 2015; Carrion et al., 2013; Clarke & Braun, 2013; Cohen et al., 2011; Kameoka et al., 2020; Mehlum et al., 2016; Shein‐Szydlo et al., 2016; Tol et al., 2014), followed by other single‐study therapeutic modalities, including trauma‐focused narrative therapy (non‐CBT) (Karibwende et al., 2023), child‐centered play therapy (Ray et al., 2020), trauma‐informed telehealth (4C) (Alfano et al., 2024), and home‐based parenting therapy (parent child interaction therapy) (Abrahamse et al., 2021). Similarly, among the psychosocial interventions, resilience/strength‐based programs were the most common (6/21; 28.6%) (Cheung & Yip, 2012; Fraser & Pakenham, 2008; Kassam‐Adams et al., 2011; Lu et al., 2021; Tam et al., 2020; van Westrhenen et al., 2019), followed by other single‐study psychosocial approaches, including family/community‐based programs (Kogan et al., 2023), a web‐based interactive program (Boring et al., 2015), and reciprocal peer tutoring (Fantuzzo et al., 2005). Detailed intervention descriptions are provided in Supporting Information S2: Appendix S3.

### Risk of bias

Across 21 trials, the overall risk of bias was low in 11 studies (52.4%) and had some concerns in 10 studies (47.6%). No study was rated high risk. For depression (*k* = 6), half were rated as some concerns (3/6, 50.0%), while the remaining half were rated as low risk (3/6, 50.0%). For anxiety (*k* = 6), most were low risk (4/6, 66.7%), and the remaining studies had some concerns (2/6, 33.3%). For PTSD/psychological distress (*k* = 8), the majority had some concerns (5/8, 62.5%), with the remainder low risk (3/8, 37.5%). For combined anxiety + depression outcomes (*k* = 4), ratings were evenly split between some concerns (2/4, 50.0%) and low risk (2/4, 50.0%). For psychosocial outcomes (*k* = 13), just over half were low risk (7/13, 53.8%), and the remaining studies had some concerns (6/13, 46.2%) (see Supporting Information S2: Appendix S3). Publication bias was not assessed because each meta‐analysis included fewer than 10 studies (Higgins, 2023; Jakobsen et al., 2014).

### Meta‐analysis of interventions among children and adolescents with documented ACE exposure and anxiety

The meta‐analysis of six RCTs examining CBT, along with trauma‐focused and resilience‐building interventions showed a statistically significant reduction in anxiety symptoms compared with control conditions (*k* = 6; Hedges' *g* = −0.46; 95% CI: −0.86 to −0.06; *I* ^2^ = 66.5%, *p* = 0.0108) (Carrion et al., 2013; Cohen et al., 2011; Kameoka et al., 2020; Karibwende et al., 2023; Lu et al., 2019; Shein‐Szydlo et al., 2016) (see Figure 2). The subgroup analysis by intervention modality showed a significant reduction in anxiety symptoms for trauma‐focused interventions (*k* = 2; Hedges' *g* = −0.90; 95% CI: −1.66 to −0.14; *I* ^2^ = 71.4%, *p* = 0.0614) (see Figure 2). However, no significant effect was observed for CBT/variants (*k* = 3; Hedges' *g* = −0.13; 95% CI: −0.44 to 0.18; *I* ^2^ = 0.0%, *p* = 0.7427) or resilience/strength‐based interventions (*k* = 1; Hedges' *g* = −0.26; 95% CI: −0.83 to 0.31). Between‐modality differences were not statistically significant (*χ* ^2^ = 3.36, df = 2, *p* = 0.186).

**FIGURE 2:**
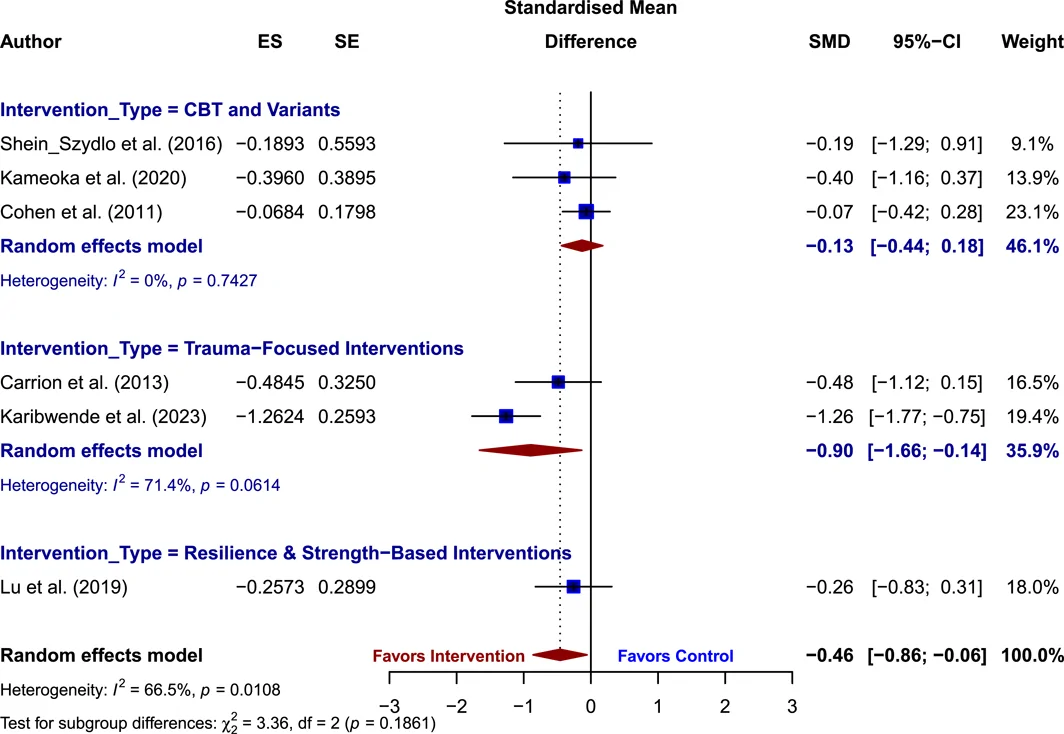
Anxiety outcomes by intervention modality: Subgroup meta‐analysis of CBT/variants, trauma‐focused, and resilience/strength‐based interventions. CBT, cognitive behavioral therapy.

When stratified by country income level, pooled effects were small and non‐significant in high‐income (*k* = 3; Hedges' *g* = −0.20; 95% CI: −0.50 to 0.09; *I* ^2^ = 0.0%, *p* = 0.4595) and upper‐middle‐income settings (*k* = 2; Hedges' *g* = −0.24; 95% CI: −0.75 to 0.26; *I* ^2^ = 0.0%, *p* = 0.9141), while a single low ‐income study showed a large, significant benefit (*k* = 1; Hedges' *g* = −1.26; 95% CI: −1.77 to −0.75). Subgroup differences were significant overall (*χ* ^2^ = 13.09, df = 2, *p* = 0.0014) (see Figure 3).

**FIGURE 3:**
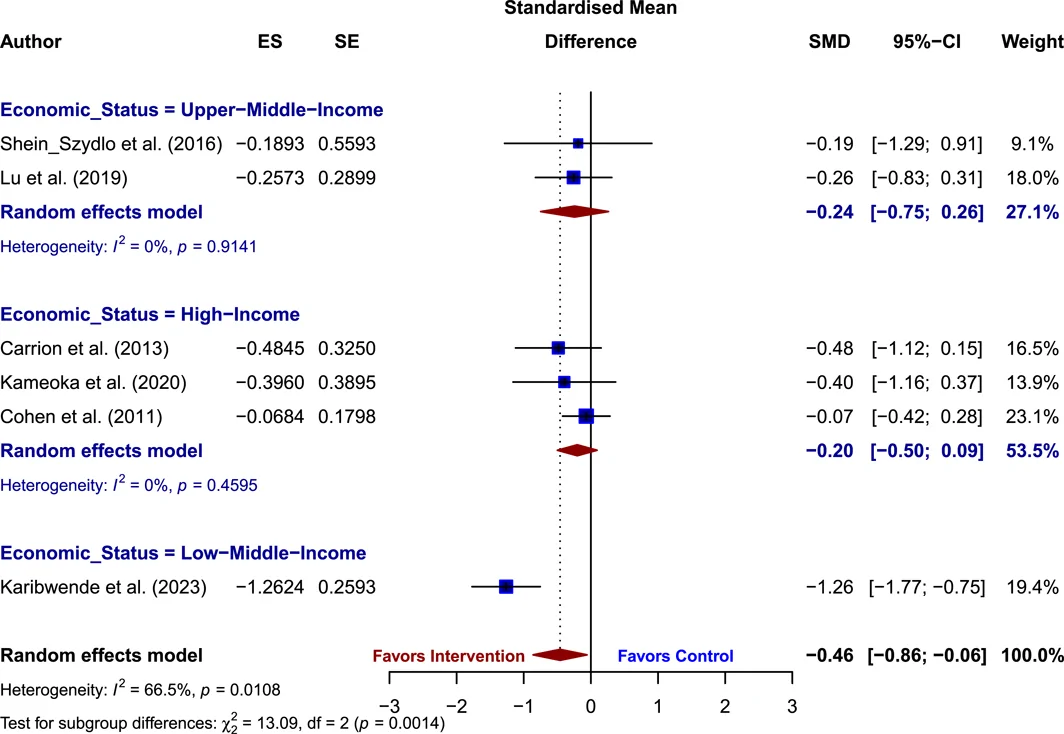
Anxiety outcomes by country income level: Subgroup meta‐analysis for high‐, upper‐middle, and lower‐middle‐income settings (World Bank classification).

In subgroup analyses stratified by reported ACE category, studies involving socio‐environmental adversity only showed no statistically significant effect (*k* = 3; Hedges' *g* = −0.59; 95% CI: −1.30 to 0.12; *I* ^2^ = 86.0%, *p* = 0.0008). Single‐study strata, maltreatment only (*k* = 1; Hedges' *g* = −0.19; 95% CI: −1.29 to 0.91), maltreatment + socio‐environmental trauma (*k* = 1; Hedges' *g* = −0.40; 95% CI: −1.16 to 0.37), and maltreatment + household dysfunction (*k* = 1; Hedges' *g* = −0.26; 95% CI: −0.83 to 0.31), also showed no significant effects. Subgroup differences by trauma type were not significant (*χ* ^2^ = 0.63, df = 3, *p* = 0.8888).

### Meta‐analysis of interventions among children and adolescents with documented ACE exposure and depression

The meta‐analysis of 6 RCTs did not yield a statistically significant pooled effect on depressive symptoms among children and young people exposed to ACEs (*k* = 6; Hedges' *g* = −0.12; 95% CI: −0.29 to 0.05; *I* ^2^ = 48.7%, *p* = 0.0826) (see Figure 4). Moderate‐study heterogeneity was observed and explored via subgroup analyses by intervention duration, economic setting, delivery setting, and reported ACE exposure characteristics.

**FIGURE 4:**
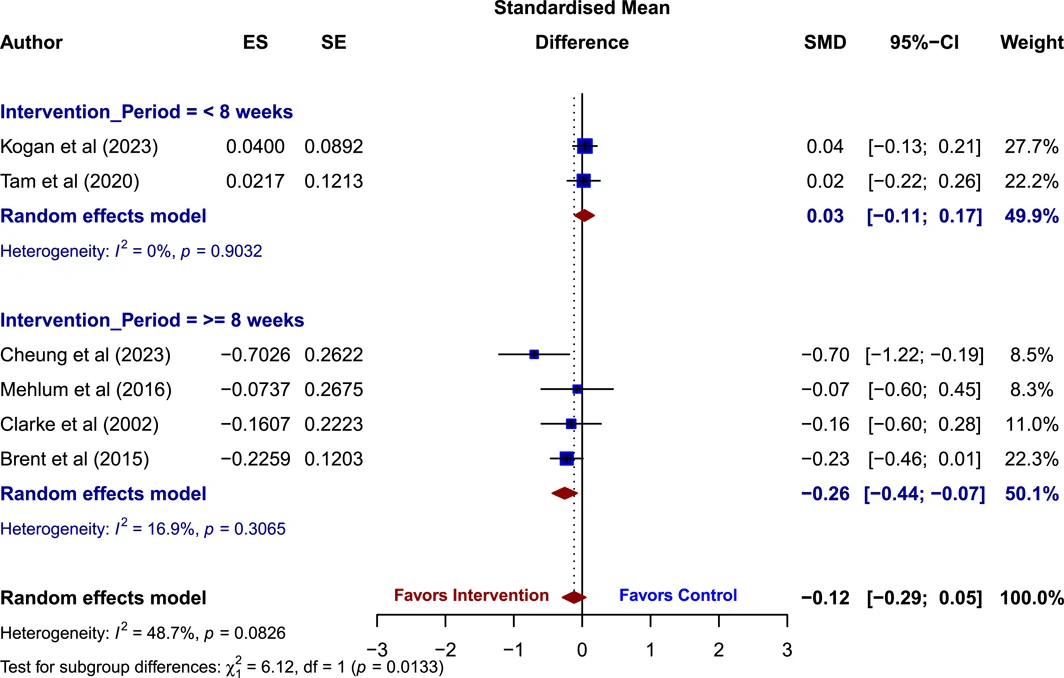
Depression outcomes stratified by intervention period: Subgroup meta‐analysis of trials with <8 weeks versus ≥8 weeks duration.

The meta‐analysis of four RCTs evaluating CBT and resilience‐building interventions lasting ≥8 weeks showed a significant reduction in depressive symptoms (*k* = 4; Hedges' *g* = −0.26; 95% CI: −0.44 to −0.07; *I* ^2^ = 16.9%, *p* = 0.3065) (see Figure 4). However, interventions lasting < 8 weeks showed no significant effect (*k* = 2; Hedges' *g* = 0.03; 95% CI: −0.11 to 0.17; *I* ^2^ = 0.0%, *p* = 0.9032). The subgroup difference was statistically significant (*χ* ^2^ = 6.12, df = 1, *p* = 0.013).

The subgroup analysis by cumulative ACE exposure level found significant differences across strata (*χ* ^2^ = 6.31, df = 2, *p* = 0.043) (see Figure 5). Studies involving participants with a higher number of reported ACE categories showed no significant effect (*k* = 4; Hedges' *g* = −0.08; 95% CI: −0.26 to 0.09; *I* ^2^ = 10.8%, *p* = 0.3392). One study with moderate cumulative ACE exposure (*k* = 1) also showed no significant effect, whereas one study with lower cumulative ACE exposure demonstrated a large and significant reduction on depression symptoms (*k* = 1; Hedges' *g* = −0.70; 95% CI: −1.22 to −0.19).

**FIGURE 5:**
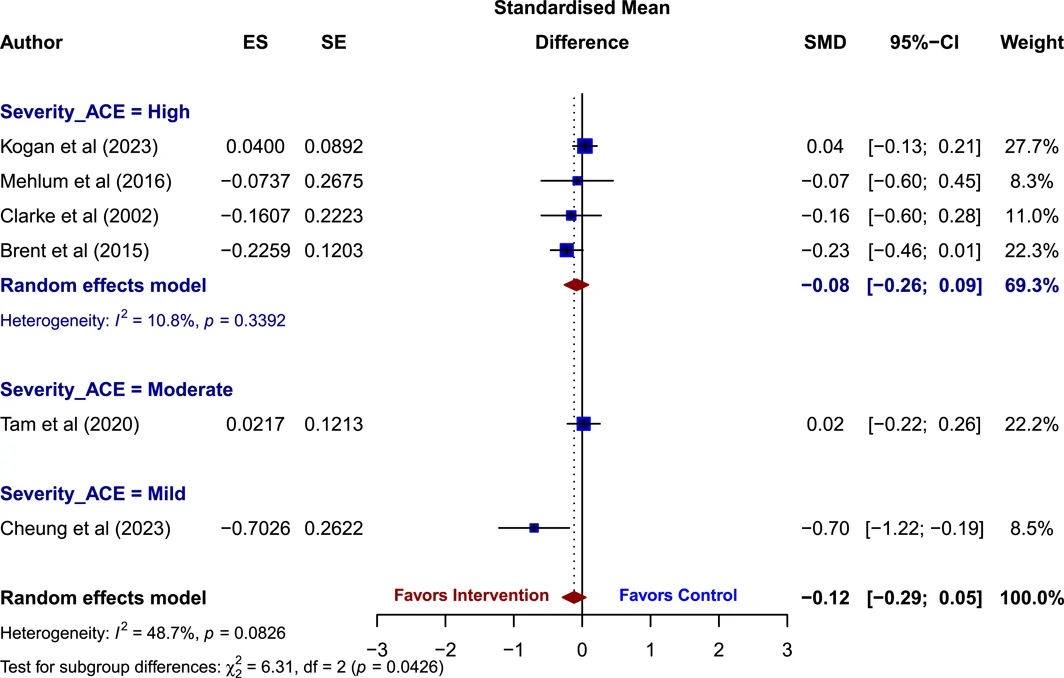
Depression outcomes by ACE severity level: Subgroup meta‐analysis for high, moderate, and mild severity strata. ACE, Adverse Childhood Experience.

The subgroup analysis by reported ACE category found significant heterogeneity across categories (*χ* ^2^ = 9.88, df = 4, *p* = 0.048). Interventions targeting socioeconomic adversity showed a large and significant benefit (*k* = 1; Hedges' *g* = −0.70; 95% CI: −1.22 to −0.19), while parental‐depression interventions suggested a borderline effect (*k* = 2; Hedges' *g* = −0.21; 95% CI: −0.42 to 0.00), indicating a borderline benefit of interventions among children exposed to parental depression (see Supporting Information S1: Figure S1). In contrast, interventions addressing racial discrimination (*k* = 1), migration‐related challenges (*k* = 1), and emotional abuse (*k* = 1) showed no significant effect.

The subgroup analysis by trauma cluster found that socio‐environmental‐only (*k* = 2) and complex‐trauma interventions (*k* = 2) showed no significant effect, while household dysfunction interventions suggested a small borderline effect (*k* = 2; Hedges' *g* = −0.21; 95% CI: −0.42 to 0.00; *I* ^2^ = 0.0%, *p* = 0.7963) (see Supporting Information S1: Figure S2). Subgroup differences were not statistically significant (*χ* ^2^ = 0.26, df = 2, *p* = 0.88).

Subgroup analysis by economic status revealed no significant effects in either high‐income settings (*k* = 3; Hedges' *g* = −0.08; 95% CI: −0.26 to 0.09; *I* ^2^ = 10.8%, *p* = 0.3392) or upper‐middle‐income settings (*k* = 3; Hedges' *g* = −0.30; 95% CI: −1.01 to 0.40; *I* ^2^ = 84.1%, *p* = 0.0122), with no evidence of subgroup differences (*χ* ^2^ = 0.35, df = 1, *p* = 0.56). Similarly, community‐based interventions showed no significant effect (*k* = 3; Hedges' *g* = −0.29; 95% CI: −1.01 to 0.43; *I* ^2^ = 86.1%, *p* = 0.0073), school‐based interventions (*k* = 1) also showed no significant effect, while health‐facility interventions suggested a borderline reduction on depression symptoms (*k* = 2; Hedges' *g* = −0.19; 95% CI: −0.39 to 0.00; *I* ^2^ = 0.0%, *p* = 0.8629) (see Supporting Information S1: Figure S3). The subgroup difference was not statistically significant (*χ* ^2^ = 2.12, df = 2, *p* = 0.35).

### Meta‐analysis of interventions among children and adolescents with documented ACE exposure and PTSD and psychosocial outcomes

Across the included RCTs, interventions among children and adolescents with documented ACE exposure did not produce a statistically significant pooled reduction in PTSD symptoms compared with controls (see Supporting Information S1: Figure S4). By intervention modality (e.g., CBT/variants, trauma‐focused, resilience/strength‐based), by country income level, by setting (community, school, health facility), and by reported ACE category, pooled subgroup effects were non‐significant, and tests for subgroup differences were non‐significant where estimable. Overall, no significant effect was observed on PTSD symptoms across analyses.

For broader psychosocial functioning and adjustment (domains including emotional regulation, coping, interpersonal functioning, quality of life, parenting, and global functioning), pooled analyses showed no statistically significant effect of interventions among children and adolescents with documented ACE exposure (see Supporting Information S1: Figure S5). Subgroup analyses—by intervention modality, country income level, setting, duration (where data allowed), and reported ACE category—were non‐significant, and tests for subgroup differences were non‐significant.

## DISCUSSION

This systematic review and meta‐analysis synthesized evidence on the effectiveness of psychosocial and therapeutic interventions delivered to children and adolescents aged <18 years with documented ACE exposure. The pooled analysis demonstrated that TF‐CBT and resilience building interventions significantly reduced anxiety symptoms, with the greatest benefits observed for trauma‐focused interventions and those implemented in low‐ and middle‐income countries (LMICs). Cognitive behavioral therapy and resilience building interventions lasting longer than 8 weeks and those addressing specific ACE‐related contents, such as socioeconomic adversity, showed meaningful improvements in depressive symptoms. In contrast, stand‐alone CBT with shorter or less intensive models, demonstrated limited benefits.

Our results are consistent with a previous review on trauma‐informed frameworks, which showed that trauma‐focused intervention models, particularly TF‐CBT, are among the most effective in improving mental health outcomes across diverse patient groups (Chin et al., 2024). Trauma‐informed approaches may be particularly beneficial for children with ACE exposure because they explicitly address hyperarousal, mistrust, and difficulties with emotional regulation that can arise from adverse or traumatic experiences, rather than targeting symptoms in isolation (Substance Abuse and Mental Health Services Administration, 2014). By emphasizing safety, predictability, and collaborative engagement, trauma‐informed interventions may address some of the relational and physiological sequelae through which ACEs are thought to contribute to poorer mental health outcomes, even among children who do not meet full diagnostic criteria for a trauma‐related disorder (Nelson et al., 2020).

Importantly, our study extends this evidence by demonstrating that, among children and adolescents with documented ACE exposure and mental health needs, trauma‐informed interventions, as well as generic CBT and resilience‐building programs lasting ≥8 weeks, may lead to improved mental health outcomes. The greater effectiveness of interventions lasting ≥8 weeks may reflect the additional time needed to build therapeutic trust and engagement, particularly for children with a history of interpersonal adversity who may be more cautious in forming new relationships (Asseffa et al., 2026). Longer interventions may also allow more opportunity for skill consolidation, such as emotional regulation or coping strategies, which typically require repeated practice and reinforcement rather than one‐off instruction (Cibralic et al., 2022; Klein et al., 2020; Nakao et al., 2021).

These findings do not necessarily indicate that ACE exposure requires a distinct therapeutic modality. Rather, they suggest that an individual’s trauma history should be considered when tailoring intervention delivery, engagement strategies, and therapeutic goals, particularly for children and adolescents exposed to adversity (Asseffa et al., 2026; Giri et al., 2025).

The observed heterogeneity may also reflect differences in the nature and timing of ACE exposure, participant characteristics, baseline symptom severity, intervention intensity, and whether interventions explicitly incorporated trauma‐informed principles (Lee & Rawlings, 2022; Pearce et al., 2019; Sanders et al., 2020). Therefore, our findings should be interpreted as reflecting intervention effectiveness within populations with ACE exposure rather than evidence that ACE exposure independently modifies treatment effects. Notably, for children and adolescents whose presenting difficulties are closely linked with trauma‐related experiences, interventions that explicitly incorporate trauma‐focused components may offer additional benefits, particularly when combined with broader strategies that strengthen coping, relationships, and resilience (Cohen et al., 2004; Goldbeck et al., 2016; Gone et al., 2019; Goodkind et al., 2012; Jongen et al., 2023; Kameoka et al., 2020; Thielemann et al., 2022).

The lack of significant pooled effects for depression, PTSD or broader psychosocial outcomes does not necessarily indicate that interventions are ineffective for children and adolescents with ACE exposure. Rather, it may reflect variability in intervention content, delivery approaches, participant characteristics, and outcome measurement (Asseffa et al., 2026). For the children and young people with documented ACE exposure, sustained engagement and therapeutic continuity may be particularly important for building trust and supporting skill development (Cibralic et al., 2022; Klein et al., 2020; Nakao et al., 2021). Short‐term or single component programs seem to have limited capacity to influence complex and multifactorial mental health difficulties, particularly where children present with multiple psychosocial stressors or ongoing adversity (Asseffa et al., 2026).

In this view, future interventions may need to integrate family, school, and community components, or adopt multi‐level models (e.g., CBT, trauma‐focused strategies, resilience‐strengthening modules) to impact wider psychosocial outcomes. Consistent with trauma‐informed care principles, interventions may need to consider not only symptom reduction but also safety, trust, empowerment, caregiver involvement, and the broader social context in which children and adolescents receive care (Substance Abuse and Mental Health Services Administration, 2014).

In the present systematic review and meta‐analysis, neither community‐ nor school‐based interventions showed significant pooled effects, whereas health facility–based interventions demonstrated a borderline improvement in depressive symptoms. Differences in privacy, confidentiality, perceived safety, therapeutic relationships, and continuity of care across settings may partly explain variation in intervention effects (Kafka et al., 2024). This pattern also suggests that the delivery setting alone does not determine effectiveness. The quality and intensity of implementation, along with consideration of multiple psychosocial factors, such as mood, personality, behavior, experiences of racism, culture, family and peer relationships, and broader socioeconomic and contextual influences, are likely key to achieving meaningful outcomes among younger populations (Huey et al., 2023).

The meta‐analysis of one RCT conducted in Rwanda showed greater benefits on anxiety symptoms in lower‐middle‐income country settings (Karibwende et al., 2023). This may reflect the importance of stronger community engagement, higher baseline symptom severity (allowing larger effect sizes), or greater contextual sensitivity of interventions in such settings. Contextually adapted, community‐embedded mental health strategies, may be effective and relevant for children and adolescents particularly in resource‐limited settings (Brent et al., 2015; Carrion et al., 2013; Clarke & Braun, 2013; Cohen et al., 2011; Kameoka et al., 2020; Mehlum et al., 2016; Shein‐Szydlo et al., 2016; Tol et al., 2014). However, this finding was based on a single RCT and should not be interpreted as evidence of greater intervention effectiveness in LMIC settings overall.

### Strengths and limitations

This systematic review and meta‐analysis provides a comprehensive synthesis of psychosocial and therapeutic interventions targeting children and adolescents with documented ACE exposure across a broad spectrum of mental health and psychosocial outcomes.

However, our systematic review has some limitations. First, the relatively small number of included RCTs limited our ability to conduct more detailed subgroup analyses, including gender, socioeconomic status, and other intersectional factors, thereby restricting insights into equity, accessibility, and differential effectiveness. Moreover, the subgroup analyses that were conducted were often based on very small numbers of studies (*k* = 1–3) and were therefore substantially underpowered. These findings should be regarded as exploratory and hypothesis‐generating rather than conclusive. Second, most included studies originated from high‐income countries, with limited data from LMICs, where ACEs are highly prevalent, and access to mental health services remains constrained. This imbalance underscores the need for locally adapted, culturally safe, and resource‐sensitive intervention studies across LMIC settings.

Third, substantial clinical and methodological heterogeneity across intervention types, outcome measures, and follow‐up durations reduced comparability across studies, and may have contributed to variability in effect estimates. Comparator conditions varied considerably across studies, including usual care, standard services, and wait‐list controls, which may have contributed to heterogeneity in the pooled estimates. Additionally, studies included in this meta‐analysis varied in how ACE exposure was defined and assessed, ranging from single‐item indicators of specific adversities (e.g., orphan status, exposure to armed conflict) (Bethell et al., 2017) to broader, multi‐item ACE inventories (Felitti et al., 1998; World Health Organization, 2018). This variation in measurement approach may have contributed to the observed heterogeneity across studies and should be considered when interpreting pooled findings.

Fourth, we restricted inclusion to studies published in English from 2000 onwards, which may have introduced language and time‐period selection bias and limited the generalizability of findings. Furthermore, conventional ACE measures may not fully capture culturally or contextually specific adversities experienced by populations such as refugee and conflict‐affected children (Abdelhamid et al., 2024). This is an important consideration for the broader applicability of ACE‐informed interventions and for future research in specific populations. Fifth, publication bias could not be formally assessed because fewer than 10 studies contributed to each meta‐analysis. Therefore, the possibility of publication bias cannot be excluded.

Finally, although a statistically significant pooled effect was observed for anxiety, this finding should be interpreted cautiously given the small number of contributing studies and the moderate level of between‐study heterogeneity. Sensitivity analyses demonstrated that the pooled effect remained statistically significant after exclusion of the study with the largest effect size (Karibwende et al., 2023), supporting the robustness of the finding. Nevertheless, future intervention research should prioritize adequately powered RCTs that incorporate standardized measures of ACE exposure and mental health outcomes, distinguish between prevention‐ and treatment‐oriented interventions, and evaluate whether trauma‐informed approaches improve engagement and clinical outcomes among children and adolescents with documented ACE exposure.

## CONCLUSION

Trauma‐focused interventions showed significant pooled effects in reducing anxiety symptoms, whereas improvements in depressive symptoms were also evident in generic CBT and resilience‐building interventions lasting ≥8 weeks and in studies involving participants with lower cumulative ACE exposure or socioeconomic adversity. Shorter or less intensive interventions generally demonstrated limited or inconsistent effects. Evidence for PTSD and broader psychosocial outcomes remains inconclusive. Integrating trauma‐informed, culturally responsive, and contextually appropriate approaches into child and adolescent mental health services may enhance engagement and support effective care for young people with documented ACE exposure.

## AUTHOR CONTRIBUTIONS

**Meless G. Bore**: Conceptualization; formal analysis; methodology; writing—original draft; writing—review and editing. **Julaine Allan**: Writing—review and editing. **Setognal B. Aychiluhm**: Formal analysis; methodology; software; visualization; writing—review and editing. **Kedir Y. Ahmed**: Writing—review and editing. **Abel Fekadu Dadi**: Writing—review and editing. **Elsa Awoke Fentie**: Writing—review and editing. **Subash Thapa**: Conceptualization; formal analysis; methodology; project administration; supervision; visualization; writing—original draft; writing—review and editing.

## CONFLICT OF INTEREST STATEMENT

The authors declare no conflicts of interest.

## ETHICAL CONSIDERATIONS

Not applicable. Ethical approval was not required as this study involved secondary analysis of published data only.

## Supporting information

Supporting Information S1

Supporting Information S2

## Data Availability

The data that supports the findings of this study are available in the article and its supplementary material.
