# Supplementary material for: Effectiveness of psychosocial and therapeutic interventions for children and adolescents with adverse childhood experiences: A systematic review and meta‐analysis
Source: JCPP Adv. 2026 Nov 4:e70186. Online ahead of print. doi: 10.1002/jcv2.70186 (PMC13734392; doi:10.1002/jcv2.70186)
Supplement: Supplementary file 1 [file JCV2-9999-e70186-s001.docx]

**Effectiveness of psychosocial and therapeutic interventions for children and adolescents with adverse childhood experiences: A systematic review and meta-analysis**

**Supporting Information**

 **PRISMA 2020 Checklist**

**Appendix S1. PRISMA 2020 Checklist**

| **Section and Topic** | **Item #** | **Checklist item** | **Location where item is reported** |
| --- | --- | --- | --- |
| **TITLE** | | |  |
| Title | 1 | Identify the report as a systematic review. | Page 1 |
| **ABSTRACT** | | |  |
| Abstract | 2 | See the PRISMA 2020 for Abstracts checklist. | Pages 2-3 |
| **INTRODUCTION** | | |  |
| Rationale | 3 | Describe the rationale for the review in the context of existing knowledge. | Pages 4-7 |
| Objectives | 4 | Provide an explicit statement of the objective(s) or question(s) the review addresses. | Pages 7 |
| **METHODS** | | |  |
| Eligibility criteria | 5 | Specify the inclusion and exclusion criteria for the review and how studies were grouped for the syntheses. | Page 7-9 |
| Information sources | 6 | Specify all databases, registers, websites, organisations, reference lists and other sources searched or consulted to identify studies. Specify the date when each source was last searched or consulted. | Page 9 |
| Search strategy | 7 | Present the full search strategies for all databases, registers and websites, including any filters and limits used. | Appendix S2 |
| Selection process | 8 | Specify the methods used to decide whether a study met the inclusion criteria of the review, including how many reviewers screened each record and each report retrieved, whether they worked independently, and if applicable, details of automation tools used in the process. | Pages 9-10 |
| Data collection process | 9 | Specify the methods used to collect data from reports, including how many reviewers collected data from each report, whether they worked independently, any processes for obtaining or confirming data from study investigators, and if applicable, details of automation tools used in the process. | Pages 9-10 |
| Data items | 10a | List and define all outcomes for which data were sought. Specify whether all results that were compatible with each outcome domain in each study were sought (e.g. for all measures, time points, analyses), and if not, the methods used to decide which results to collect. | Pages 9-11 |
|  | 10b | List and define all other variables for which data were sought (e.g. participant and intervention characteristics, funding sources). Describe any assumptions made about any missing or unclear information. | Appendix S3 |
| Study risk of bias assessment | 11 | Specify the methods used to assess risk of bias in the included studies, including details of the tool(s) used, how many reviewers assessed each study and whether they worked independently, and if applicable, details of automation tools used in the process. | Page 10 |
| Effect measures | 12 | Specify for each outcome the effect measure(s) (e.g. risk ratio, mean difference) used in the synthesis or presentation of results. | Appendix S3 |
| Synthesis methods | 13a | Describe the processes used to decide which studies were eligible for each synthesis (e.g. tabulating the study intervention characteristics and comparing against the planned groups for each synthesis (item #5)). | Appendix S3 |
|  | 13b | Describe any methods required to prepare the data for presentation or synthesis, such as handling of missing summary statistics, or data conversions. | Page 10 |
|  | 13c | Describe any methods used to tabulate or visually display results of individual studies and syntheses. | Pages 10-12 |
|  | 13d | Describe any methods used to synthesize results and provide a rationale for the choice(s). If meta-analysis was performed, describe the model(s), method(s) to identify the presence and extent of statistical heterogeneity, and software package(s) used. | Page 10-12 |
|  | 13e | Describe any methods used to explore possible causes of heterogeneity among study results (e.g. subgroup analysis, meta-regression). | Page 11 |
|  | 13f | Describe any sensitivity analyses conducted to assess robustness of the synthesized results. | Pages 11-12 |
| Reporting bias assessment | 14 | Describe any methods used to assess risk of bias due to missing results in a synthesis (arising from reporting biases). | Page 10 |
| Certainty assessment | 15 | Describe any methods used to assess certainty (or confidence) in the body of evidence for an outcome. | Page 10-12 |
| **RESULTS** | | |  |
| Study selection | 16a | Describe the results of the search and selection process, from the number of records identified in the search to the number of studies included in the review, ideally using a flow diagram. | Page 12 |
|  | 16b | Cite studies that might appear to meet the inclusion criteria, but which were excluded, and explain why they were excluded. | Figure 1 |
| Study characteristics | 17 | Cite each included study and present its characteristics. | Page 13 |
| Risk of bias in studies | 18 | Present assessments of risk of bias for each included study. | Pages 13-14 |
| Results of individual studies | 19 | For all outcomes, present, for each study: (a) summary statistics for each group (where appropriate) and (b) an effect estimate and its precision (e.g. confidence/credible interval), ideally using structured tables or plots. | Pages 12-17 |
| Results of syntheses | 20a | For each synthesis, briefly summarise the characteristics and risk of bias among contributing studies. | Pages 13-14 |
|  | 20b | Present results of all statistical syntheses conducted. If meta-analysis was done, present for each the summary estimate and its precision (e.g. confidence/credible interval) and measures of statistical heterogeneity. If comparing groups, describe the direction of the effect. | Figures 2-5, Figures S1-S5, Pages 14-17 |
|  | 20c | Present results of all investigations of possible causes of heterogeneity among study results. | Pages 12-17 |
|  | 20d | Present results of all sensitivity analyses conducted to assess the robustness of the synthesized results. | Figures S1-S5 |
| Reporting biases | 21 | Present assessments of risk of bias due to missing results (arising from reporting biases) for each synthesis assessed. | Pages 13-14 |
| Certainty of evidence | 22 | Present assessments of certainty (or confidence) in the body of evidence for each outcome assessed. | Figures 2-5; Pages 12-17 |
| **DISCUSSION** | | |  |
| Discussion | 23a | Provide a general interpretation of the results in the context of other evidence. | Pages 17-21 |
|  | 23b | Discuss any limitations of the evidence included in the review. | Pages 21-23 |
|  | 23c | Discuss any limitations of the review processes used. | Page 21-23 |
|  | 23d | Discuss implications of the results for practice, policy, and future research. | Page 21 |
| **OTHER INFORMATION** | | |  |
| Registration and protocol | 24a | Provide registration information for the review, including register name and registration number, or state that the review was not registered. | PROSPERO (CRD420251032622), Page 25 |
|  | 24b | Indicate where the review protocol can be accessed, or state that a protocol was not prepared. | Page 25 |
|  | 24c | Describe and explain any amendments to information provided at registration or in the protocol. | Pages 7-9 |
| Support | 25 | Describe sources of financial or non-financial support for the review, and the role of the funders or sponsors in the review. | Page 24 |
| Competing interests | 26 | Declare any competing interests of review authors. | Page 24 |
| Availability of data, code and other materials | 27 | Report which of the following are publicly available and where they can be found: template data collection forms; data extracted from included studies; data used for all analyses; analytic code; any other materials used in the review. | Appendix S3 |

*From:* Page MJ, McKenzie JE, Bossuyt PM, Boutron I, Hoffmann TC, Mulrow CD, et al. The PRISMA 2020 statement: an updated guideline for reporting systematic reviews. BMJ 2021;372:n71. doi: 10.1136/bmj.n71. This work is licensed under CC BY 4.0. To view a copy of this license, visit <https://creativecommons.org/licenses/by/4.0/>

**Appendix S2. Search terms and Boolean operators**

**Appendix S3. Data for Meta-analysis and Quality appraisal**

Provided in a Separate file

**Line 1**: "children" OR "adolescent" OR "young people" OR "people under 19 years")
**AND**
**Line 2**: ("personalized treatment" OR "individualized therapy" OR "tailored intervention" OR “intervention” OR “Therapy” OR “Mental health treatment” OR “Mental Health Care” OR “Individual care” OR "Trauma-Focused Cognitive Behavioral Therapy" OR "TF-CBT" OR "Dialectical Behavior Therapy" OR "DBT" OR "Mindfulness-Based Cognitive Therapy" OR "MBCT" OR "family-based intervention" OR "personalized medication management")
**AND**
**Line 3**: ("Standard care" OR "usual care" OR “no treatment” OR “Other Treatment” OR "peer support" OR "social skills training" OR "pharmacological treatment" OR "waitlist control")
**AND**
**Line 4**: ("Psychosocial well-being" OR "Mental health" OR “Mental health symptoms” OR “Improvement” OR “Mental health improvement” OR “Psychosocial outcomes” OR "Social functioning" OR "Quality of life" OR "Emotional resilience")
**AND**
**Line 5**: (“Adverse Childhood Experiences” OR “ACEs” OR “Child abuse” OR “Physical abuse” OR “Psychological abuse” OR “Emotional abuse” OR “Sexual abuse” OR “Neglect” OR “Trauma” OR “Childhood trauma” OR “Co-occurring mental disorders” OR “Mental health disorder” OR “Mental illness” OR “Psychological distress” OR “Maltreatment” OR “Household dysfunction” OR “Early life stress” OR “Depression” OR “Anxiety” OR “PTSD” OR “Psychiatric disorder” OR “Obsessive-compulsive disorder” OR “OCD” OR “Eating disorder” OR “Anorexia Nervosa” OR “Bulimia Nervosa” OR “Attention-deficit hyperactivity disorder” OR “ADHD” OR “Bipolar disorder” OR “Schizophrenia” OR “Personality disorder” OR “Borderline Personality Disorder” OR “Borderline disorder” OR “Suicide” OR “Suicidal ideation” OR “Self-harm” OR “Parent substance use” OR “Parent alcohol use” OR “Parent drug use” OR “Parent conflict” OR “Parent separation” OR “Parent divorce” OR “Parental neglect” OR “Peer violence”)

**Figure S1.** Depression outcomes by ACE type: subgroup meta-analysis for socioeconomic adversity, parental depression, racial discrimination, migration-related challenges, and emotional abuse.

**Figure S2.** Depression outcomes by trauma clusters: subgroup meta-analysis for socio-environmental only, household dysfunction, and complex trauma categories.

**Figure S3.** Depression outcomes by intervention setting: subgroup meta-analysis for community-, school-, and health facility–based interventions.

**Figure S4.** Pooled effect on post-traumatic stress disorder and distress symptoms in children and youth exposed to ACEs: random-effects meta-analysis of eight RCTs (negative SMD favors intervention).

**Figure S5.** Pooled effect on psychosocial problems in children and youth exposed to ACEs: random-effects meta-analysis of thirteen RCTs (negative SMD favors intervention).
